# Plant-Derived Compounds: A Promising Tool for Dental Caries Prevention

**DOI:** 10.3390/cimb46060315

**Published:** 2024-05-26

**Authors:** Konstantinos Tzimas, Maria Antoniadou, Theodoros Varzakas, Chrysoula (Chrysa) Voidarou

**Affiliations:** 1 Department of Operative Dentistry, National and Kapodistrian University of Athens, 11521 Athens, Greece; kwstastzimas@dent.uoa.gr; 2 Department of Food Science and Technology, University of the Peloponnese, 24100 Kalamata, Greece; t.varzakas@uop.gr; 3 Department of Agriculture, University of Ioannina, 47132 Arta, Greece

**Keywords:** plant-derived compounds, natural extracts, traditional remedies, oral health, dental caries prevention, antimicrobial properties, sustainability, eco-conscious practices, natural compounds

## Abstract

There is a growing shift from the use of conventional pharmaceutical oral care products to the use of herbal extracts and traditional remedies in dental caries prevention. This is attributed to the potential environmental and health implications of contemporary oral products. This comprehensive review aims at the analysis of plant-derived compounds as preventive modalities in dental caries research. It focuses on data collected from 2019 until recently, trying to emphasize current trends in this topic. The research findings suggest that several plant-derived compounds, either aqueous or ethanolic, exhibit notable antibacterial effects against *Streptococcus mutans* and other bacteria related to dental caries, with some extracts demonstrating an efficacy comparable to that of chlorhexidine. Furthermore, in vivo studies using plant-derived compounds incorporated in food derivatives, such as lollipops, have shown promising results by significantly reducing *Streptococcus mutans* in high-risk caries children. In vitro studies on plant-derived compounds have revealed bactericidal and bacteriostatic activity against *S. mutans*, suggesting their potential use as dental caries preventive agents. Medicinal plants, plant-derived phytochemicals, essential oils, and other food compounds have exhibited promising antimicrobial activity against oral pathogens, either by their anti-adhesion activity, the inhibition of extracellular microbial enzymes, or their direct action on microbial species and acid production. However, further research is needed to assess their antimicrobial activity and to evaluate the cytotoxicity and safety profiles of these plant-derived compounds before their widespread clinical use can be recommended.

## 1. Introduction

Oral health has always been considered an inseparable part of general health since it is inextricably linked to general well-being [1]. Every aggravating factor related to oral health will affect the quality of life and the psycho-social aspects of life, directly or indirectly [2]. The direct and indirect global costs related to oral pathologies are estimated at around USD 298 and USD 144 billion, respectively [3]. Dental caries is the most prevalent non-transmissible disease and is considered a significant global public health issue [4,5]. According to the World Health Organization (WHO), oral diseases affect around 3.5 billion people worldwide, with the vast majority of people affected living in middle-income countries. Globally, approximately 2 billion people suffer from caries of permanent dentition, while 514 million children are diagnosed with caries of their primary dentition [6]. Despite continuous advancements in the field of dental care, the prevalence of dental caries remains at high levels, proving the urgent need for developing innovative preventive strategies that will complement the existing preventive armamentarium [7,8].

Dental caries is presented as a persistent, significant global oral health concern, affecting individuals of all ages, genders, and ethnicities [9,10]. It is one of the most prevalent chronic human diseases [11]. Dental caries presents a multifactorial pattern, influenced by the host, agent, and environmental factors. Carious lesions are caused by oral bacteria organized in biofilms [12]. The adherence of microorganisms commences immediately after the formation of the acquired pellicle on the surfaces of teeth and restorative–prosthetic materials [13]. The most abundant cariogenic microorganism is *Streptococcus mutans*, which produces glycosyltransferases, facilitating biofilm formation. A significant correlation has been identified between the salivary numbers of *S. mutans* and dental caries prevalence [14]. Aside from *Streptococcus mutans*, several other microorganisms, such as other Streptococcus strains, Lactobacillus, and Actinomyces species, play a pivotal role in dental caries development. These organisms produce acids that cause an abrupt decline in pH value after their interaction with dietary carbohydrates, leading to an increased risk of caries [15]. Under normal circumstances, pathogenic and physiological microorganisms exhibit a phenomenon called symbiosis, which leads to the maintenance of oral health [16]. A plethora of factors may disrupt this sensitive balance and result in dysbiosis. Inadequate oral health habits, combined with a rich low-molecular-weight carbohydrates diet, excessive amount of sugar intake, decreased fluoride exposure, reduced salivary flow, and many other factors, lead to acid formation and subsequently to tooth surface demineralization [16,17]. All in all, it is evident that the dental caries etiology is based on a four-factor concept that includes (i) an imbalance of oral microorganisms, (ii) oral environmental conditions, (iii) host response, and (iv) time [18]. While a notable decline in dental caries prevalence is present in Western developed countries in recent years [19], changes in dietary habits among younger generations, favoring refined carbohydrates, may contribute to a potential resurgence of caries incidence over time [20,21].

The confinement of the activity of these bacteria minimizes the dental caries risk. Therefore, the incorporation of fluoride in toothpaste, varnishes, water, and milk, as well as the use of pit and fissure sealants, are available tools for dental caries prevention [22,23,24,25]. Furthermore, the use of chemical agents that interfere with bacterial metabolic activities and bacterial cell adherence is a well-documented approach against biofilm development and maturation [26]. Although the daily use of fluoride may prevent dental caries and promote the remineralization of dental carious lesions, it also demonstrates drawbacks, such as fluorosis through early exposure or overexposure [27]. Antimicrobial agents such as chlorhexidine mouthwashes have been proven effective in reducing the *Streptococcus mutans* count. The mechanism of action of chlorhexidine involves disruption of the permeability barrier of bacterial cells, cytoplasmic leakage, and denaturation [28]. Due to the presence of several side effects, such as tooth staining, taste alteration, and the development of resistance of microorganisms against chemical compounds [28,29,30], the use of plant-derived compounds against cariogenic microorganisms has been thoroughly investigated [31]. The main advantage of these products is the restoration of health in a natural, non-drug-related manner, with minimum side effects and maximum efficiency. These herbs have been proven to be effective in various oral health problems such as gingivitis, periodontal disease, aphthous ulcers, halitosis, and dental caries [32]. It is therefore concluded that, although conventional therapeutic methods constitute the gold standard, natural compounds derived from foods and herbal agents have been, and deserve to be, extensively studied as newly introduced, alternative oral care products for caries prevention [33]. These compounds present interesting medical and physicochemical backgrounds that provide additional therapeutic characteristics [31]. Plant-derived compounds are preferred over conventional drugs for several reasons, such as their immense natural biocharacteristics, easier accessibility, lower cost, and wide safety spectrum. In contrast, many of the modern medicaments used in dentistry are responsible for known side effects [34].

Herbal medicine is currently used by approximately 80% of the world’s population for health-related purposes, mostly by people residing in the rural areas of developing countries [35]. Nowadays, clinical trials are investigating the effectiveness of several natural compounds in preventing dental caries [36]. Phytochemicals may serve as effective and economical treatments [37,38]. Furthermore, traditional medicinal practices (including ethnomedicine) and alternative, complementary medicine provide valuable insights into plant-derived remedies that have been used for centuries to promote oral health [39,40]. Natural compounds derived from plants and foods offer promising results in the preservation of dental health, with their antimicrobial and antioxidant properties holding the potential for controlling the risk of dental caries [41,42,43,44]. 

The multifactorial pattern of dental caries necessitates comprehensive and holistic preventive strategies that address not only the biological factors (bacteria) but also the socio-economic and environmental determinants of oral health [45]. For example, immigrants and individuals from war zones often face barriers in the level of access to dental care, leading to increased susceptibility to dental caries, due to inadequate oral hygiene practices and limited access to preventive measures. Similarly, populations affected by poverty may experience nutritional deficiencies, exacerbating their risk of dental caries. Moreover, sustainability issues highlight the importance of exploring eco-friendly and economically viable alternatives for caries prevention [46,47,48]. The integration of natural compounds into oral care protocols, the simultaneous promotion of oral health education, and the improvement of and easy access to dental services create the ideal substrate for a holistic dental caries preventive modality, especially in developing countries and vulnerable populations.

Thus, this comprehensive review aims to present in detail and criticize the existing literature regarding the use of various phytochemicals found in plant-derived compounds against dental caries. Findings from in vitro, in silico, ex vivo, in vivo, and clinical studies are combined to provide insights into the mechanisms of action, level of efficacy, and safety profiles of natural compounds for caries prevention. Finally, this study identifies the limitations of the involved studies, investigates the potential anticariogenic mechanism of action of plant-derived compounds, and highlights future perspectives for developing effective and sustainable caries prevention strategies.

## 2. Materials and Methods

**Figure 1:**
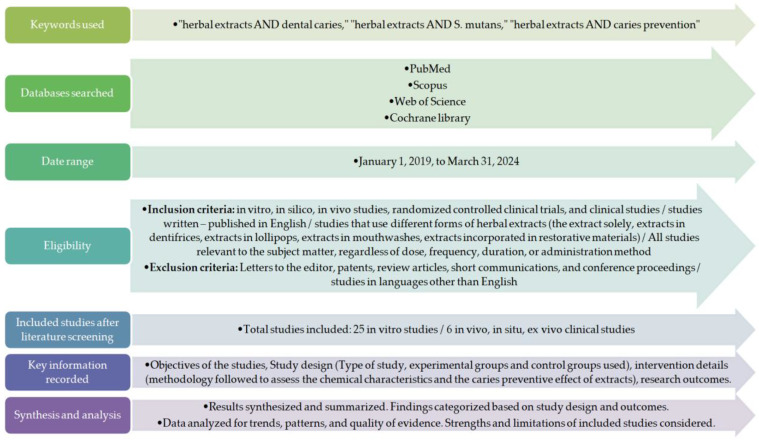
A diagram illustrating the search process for the review.

The selection process for the studies included in this review started with a comprehensive search of the following databases: PubMed, Scopus, Google Scholar, Web of Science, and Cochrane. The search strategy was developed using the following keywords: “plant-derived compounds AND dental caries”, “herbal extracts AND *Streptococcus mutans*”, and “herbal extracts AND caries prevention”. Date and language restrictions were applied. Studies in languages other than English were eliminated from the selection process. For this review, we included research published between 2019 and March 2024 to capture recent developments in the field of plant-derived compounds related to caries prevention. This date restriction has been applied since a scoping review on clinical trials with herbal products for the prevention of dental caries was published in 2019, including all the research conducted until the end of 2018 [36]. In vitro, in silico, ex vivo, in vivo studies, and randomized controlled clinical trials are included in this review article. Various product forms, such as dentifrices, chewing gum, lollipops, gels, and restorative materials, are included too. All clinical studies relevant to the subject, regardless of dose, frequency, duration, or administration method, were eligible for inclusion. Letters to the editor, patents, review articles, short communications, and conference papers were excluded. The procedure employed in this review is reflected in Figure 1.

## 3. Results

Based on the inclusion and exclusion criteria, a total of 31 studies are presented and analyzed in this comprehensive review. Table 1 records the in vitro studies conducted from 2019 until 2024 [49,50,51,52,53,54,55,56,57,58,59,60,61,62,63,64,65,66,67,68,69,70,71,72,73], and Table 2 analyzes the in vivo, in situ, and ex vivo clinical studies available in the literature [74,75,76,77,78,79]. In both tables, extensive descriptive information on the aim of the study (objectives), the type of experimental and control groups used, the methodological pattern followed, the bacterial strains chosen, and the results that emerged is present. 

### 3.1. Analysis of In Vitro Studies

Based on the data received from up-to-date in vitro studies concerning the antimicrobial activity and the effect on biofilm formation of a plethora of plant-derived compounds against oral pathogens that induce dental caries, the following significant findings need to be highlighted:

- Several plant-derived compounds, including those of licorice root, cinnamon, green tea, hibiscus, coffee pulp, and Triphala, as well as curcuma extracts, papaya extracts, honeycomb extracts, and many more plant-derived extracts, exhibited significant antimicrobial properties against various oral pathogens [49,51,54,56,59,65,66,67,72,73]. These effects were, most of the time, comparable to the effectiveness of commercially available antibacterial agents, such as chlorhexidine and fluoride mouthwashes. An ideal example is the fact that ethanolic licorice root extract demonstrated a comparable antibacterial effect to chlorhexidine mouthwash against *S. mutans*, while significantly surpassing the antimicrobial effect of aqueous licorice root extract and fluoride mouthwash [49].
- The antimicrobial activity of plant-derived compounds often showed dose-dependent responses, with higher concentrations generally leading to a greater inhibition of microbial growth and biofilm formation [56,60,61]. Caution is required when increasing the concentration of the compounds, since cytotoxic responses may appear. Research groups need to assess the biocompatibility–cytotoxicity of the different concentrations used in the experimental designs of their in vitro studies [66].
- The combination of plant-derived compounds (two, or more than two, plant-derived extracts) showed significantly greater antimicrobial results compared either to control groups (e.g., chlorhexidine) or to the independent, separate use of the investigated compounds [53,63,69,70,71]. Some herbal combinations, such as *S. striata* and *Q. infectoria* gall extracts, demonstrated synergistic effects on inhibiting the growth of cariogenic microorganisms [71]. Furthermore, *Pimpinella anisum* and *O. Vulgare* performed better regarding antimicrobial effectiveness when combined rather than when evaluated separately [63]. This finding suggests a potential for developing preventive strategies using multiple plant-derived compounds.
- Different solvents (aqueous or ethanolic) influence the potential antibacterial effect of a plant-derived compound [59,60,70,72]. For example, the ethanol extracts of coffee pulp presented superior zones of inhibition for *S. mutans* compared to aqueous extracts of coffee pulp [72]. Additionally, Balhaddad et al., in 2021, demonstrated that the type of extract and its concentration are essential factors to achieve antimicrobial effectiveness when evaluating *S. persica* as a potential dental caries preventive extract [60].
- Various phytochemicals present in plant-derived compounds contribute to their antimicrobial efficacy. These include alkaloids, flavonoids, phenols, saponins, and tannins, which are known for their antimicrobial properties. Zhang et al. in 2021 concluded that the identified phytochemicals (Ligurobustoside B, Ligurobustoside C, Ligurobustoside N, and Ligurobustoside J) accomplish the inhibition of Extracellular Polymeric Substance (EPS) synthesis and lead to the inhibition of the enzymatic activity of the Gtf proteins of *S. mutans* [57]. Furthermore, the phytochemical analysis of hibiscus extract revealed that delphinidin-3- sambubioside is identified as a particularly important inhibitory component [73]. In the research of Karnjana et al. in 2023, molecular docking revealed the evident interaction between luteolin isolated from *Cymbopogon citratus* and glucosyltransferase protein (GtfB) strengthening belief in their promising antibiofilm activity [70].

Overall, the findings from the in vitro studies suggest that herbal extracts possess significant antimicrobial and antibiofilm properties against oral pathogens, highlighting their potential use in oral healthcare products and therapies.

#### Analysis of the Methodological Pattern of the Currently Conducted In Vitro Studies

The synthesis and in-depth analysis of the available data revealed that the design of in vitro studies follows an almost identical pattern. More precisely, most studies investigating herbal extracts divide their protocol into a phytochemical analysis section and an antimicrobial analysis section. In the first section, the phytochemical profiling is based on techniques of analytical chemistry that try to separate, identify, and quantify the compounds of the herbal extracts [80,81]. The most used devices for phytochemical analysis in the up-to-date in vitro studies evaluated in this review are high-performance liquid chromatography, either solely or coupled with mass spectrometry devices [51,57,58,70,71,73], and gas chromatography coupled with mass spectrometry [53,64]. These analytical techniques allow for the identification and characterization of compounds present in the extracts. They provide information for understanding the chemical composition of the extracts and correlating specific compounds with the antimicrobial and antibiofilm activities [82,83,84]. Other less frequently used analytical techniques include thin-layer chromatography (TLC) [64], nuclear magnetic resonance (NMR) [57,58], and a combination of several tests such as Mayer’s test, Dragendorff’s test, Wagner’s test, Molish’s test, Salkowski’s test, Lieberman–Burchard’s test, the Keller Kiliani test, Ninhydrin test, Millon’s test, Ferric chloride test, Spot test, Foam test, and Saponification test [68]. Focusing on the antimicrobial analysis section of the in vitro studies, the absolute majority of the studies incorporated in their protocols the use of the agar diffusion method and the microdilution method. In the agar well diffusion method, agar plates are inoculated with a standard inoculum of the tested microorganism (mostly *S. mutans*, since this is the bacterial strain mostly investigated in the studies conducted between 2019 and 2024), and then a hole with specific diameter of 6 to 8 mm is punched aseptically with a sterile tip, and a volume (20–100 µL) of the antimicrobial agent or extract solution at the desired concentration is introduced into the well. The potential antimicrobial agent diffuses into the agar and inhibits the growth of the tested microorganism, and zones of inhibition are formed. Researchers then measure the diameter of the zone of inhibition, and comparisons between experimental groups and control groups are made [85]. Since the agar disk diffusion method is unable to quantify the amount of the antimicrobial agent diffused into the agar medium, the broth microdilution method is introduced as a tool in the antimicrobial assessment of several herbal extracts [86]. Using this technique, most researchers evaluate the minimum inhibitory concentration (MIC) of the extracts examined [49,50,51,52,53,54,56,58,60,62,63,64,66,69,70,71,72,73]. The MIC is described as the lowest concentration of an antimicrobial agent that completely inhibits the growth of the microorganism in tubes or microdilution wells, as detected by the unaided eye [87]. Viewing devices and colorimetric methods (dyes) are developed to facilitate the procedure [85]. Besides MIC determination, additional indexes are evaluated in these in vitro studies, such as the minimum bactericidal concentration (MBC) [56,58,63,70,71], minimal biofilm inhibition concentration (MBIC50) [51,54,59], and minimal biofilm reduction concentration (MBRC50) [50,51], which provide valuable information on the ability of the extracts to prevent and reduce biofilm formation, which is crucial for caries prevention. These additional indexes are not always evaluated when assessing the antimicrobial effects of plant-derived compounds against dental caries. Colony-forming unit counting (CFU/mL), combined with SEM investigations and confocal laser scanning microscopy (CLSM), is used to perform qualitative and quantitative evaluations of bacterial formation [88]. Microscopic techniques such as the use of scanning electron microscopy (SEM) and confocal laser scanning microscopy (CLSM) permit the visualization of the effects of the herbal extracts examined on bacterial colonies and biofilm structures [51,56,57,64,70]. Lastly, molecular techniques like real-time PCR (qRT-PCR) and molecular docking analysis are utilized to quantify the total bacteria and to understand the impact of the extracts on the gene expression and molecular interactions involved in bacterial inhibition and biofilm formation [57,64,70].

It should not be forgotten that in vitro studies assessing the antimicrobial effects of herbal extracts present several limitations. When conducting an in vitro study, caution should be exercised concerning the standardization of the applied procedures. Since in vitro studies use close-ended and culture-dependent methods, it is impossible to reflect intraoral conditions; therefore, more in vivo studies with appropriate study designs should be conducted.

### 3.2. Analysis of In Vivo, In Situ, and Ex Vivo Clinical Studies

A total of six (6) studies were included in this category. One study was characterized as an in situ, ex vivo study since it used volunteers who were advised to wear intraoral devices that incorporated bovine enamel slabs for a 2 h period for a biofilm to be formed. These slabs were then transferred to a laboratory environment to be treated with herbal extracts and to assess the differences in the total oral microbiome after treatment [78]. The rest of the studies may be characterized as in vivo clinical studies, since the anticaries effects of plant-derived compounds are tested on living organisms (human volunteers) in the oral environment [74,75,76,77,79]. The study designs of these in vivo studies present discrepancies regarding the number of participants, the compounds evaluated, the dosage form of the plant-derived compound, the duration of the intervention, the choice of control groups, the methodological analysis used, and the geographic origin of the research. Each clinical study evaluated different plant-derived extracts (licorice extracts, Teucrium polium extracts, miswak extracts, Emblica officinalis extracts, Rosmarinus officinalis extracts, and lastly Robusta coffee pulp extract) The number of participants also differed and ranged from 22 to 60. Two in vivo studies used high-dental-caries-risk children [74,76] (one of them used children diagnosed with Early Childhood Caries [76]). Three in vivo studies evaluated the impact of plant-derived extracts on the prevention of dental caries in children [74,76,77], and two in vivo studies used adult volunteers [75,79]. Concerning the dosage form evaluated in the studies, two in vivo protocols used plant-derived extracts as the basic component of fabricated lollipops [74,77], two other studies used the tested plant-derived compounds as constituents of a fabricated mouthwash [75,79], and one other in vivo study incorporated the plant-derived extract as part of a restorative material (GIC + miswak extracts) [76]. Some of the studies used as control groups the lack of exposure to the plant extract (placebo) [74,75,77], while some others used chlorhexidine and distilled water [76,78,79]. Regarding the bacterial strain investigated, two studies tried to evaluate and identify the total oral microbiome [74,78], whereas some other studies stuck to the perception that S. mutans is the abundant cariogenic microorganism and used it as the chosen bacterial strain [75,76,77,79]. The duration of the intervention also presents variations. The duration of the conducted in vivo studies ranges from 7 days to 3 months. The methodological patterns used are similar to those of the abovementioned in vitro studies (microdilution method, liquid chromatography, microscopy, etc.). The incorporation of a MALDI-TOF-MS (matrix-assisted laser desorption/ionization coupled to time-of-flight mass spectrometry) device for biofilm identification constitutes a novel approach in the field of the efficacy of herbal extracts in dental caries prevention [78]. The majority of the studies were conducted in India, one in China [74], and the in situ/ex vivo study in Switzerland [78]. It is therefore concluded that such protocols are mainly investigated in developing countries rather than industrial, developed regions, where access to pharmaceutical formulations is prompt. The abovementioned data highlight the need for establishing uniform, standardized protocols with a specific minimum number of participants, specific control groups, and methodological techniques for the results to be as comparable and as repeatable as possible. Focus should be given to designing proper double-blinded, randomized controlled clinical trials for the examination of plant-derived compounds’ efficacy on dental caries prevention, with an extensive duration of intervention, since this kind of research presents strong evidence quality.

## 4. Discussion

### 4.1. Limitations of the Studies

The preliminary evidence indicates that in vitro, in situ, and in vivo clinical studies for caries prevention by the use of plant-derived extracts present a heterogeneous pattern regarding design, quality, and the products evaluated. Overwhelmingly, the studies evaluated in this review reported encouraging results as to the potential antimicrobial and antibiofilm properties of herbal extracts, but since flaws and biases appear in the study designs, it is difficult to draw safe conclusions. The main flaw in in vitro studies is the culturing bias and the absence of standardization concerning the preparation method of the examined extracts. In general, extraction procedures include maceration, digestion, decoction, infusion, percolation, Soxhlet extraction, superficial extraction, and ultrasound-assisted and microwave-assisted extractions. The choice of extraction method is crucial and depends on the nature of the solvent (aqueous, ethanolic) and the intended use of the herbal extract. Different extraction methods may influence the outcome of the study of plant compounds, and some extraction methods show cost-effective, time-saving, and energy-saving characteristics [89]. Unfortunately, only one in vitro study analyzed the extraction methods of the plant-derived compounds [72]. Most of the in vitro studies solely use *Streptococcus mutans* as the chosen bacterial strain. It is frequently utilized, as it is one of the primary bacteria associated with dental caries formation [12,90,91]. Further species investigated include *S. salivarius*, *S. sanguinis*, *L. casei*, *L. acidophilus*, and *A. viscosus*. Only one study used *B. gaemokensis* [64], and few studies incorporated in their protocol the use of clinical isolates of *S. mutans* [57,59]. At this point, it should be mentioned that, although in traditional microbiology, the individual cell unit is typically the focus, in the case of dental biofilms, the whole organism is working together and each bacterium is dependent on the other species present. Therefore, typical microbiological approaches may not be sufficient for the study of biofilm-forming bacteria. Treatment strategies must incorporate a more holistic, ecological approach to the control of the dental biofilm, which is best accomplished using molecular genetic, culture-independent techniques [92]. Most studies omit to report which part of the plant is used to produce an extract. Furthermore, differences in the dosage forms in in vitro studies are present. Most of the time, herbal extracts are investigated in their primary state, whereas some researchers incorporate ethanolic or aqueous herbal extracts in mouthwashes, toothpastes, and nanoemulsion. These facts may complicate the comparison between the existing studies. Moreover, only a few studies incorporated in their study design an evaluation of the cytotoxicity of herbal extracts (biocompatibility). Further research is needed to evaluate the cytotoxicity and safety profiles of these herbal extracts before widespread clinical use can be recommended. Lastly, it should not be forgotten that in vitro studies fail to simulate to the greatest extent intraoral conditions, a fact that decreases their evidence power. Some limitations concerning in vivo clinical studies coincide with those mentioned for in vitro research. The greatest limitation has to do with the duration of the intervention. Focusing on Table 2, it is concluded that the duration of the conducted in vivo studies ranged from 7 days to 3 months [74,75,76,77,78,79]. The major drawback of the short periods of the interventions is that, even if a strong positive effect is shown, the capability of that specific product to maintain the preventive effect in the long term remains questionable [36]. The number of volunteers participating in in vivo studies and the suitable selection of control groups play a pivotal role in the clinical credibility and statistical reliability of the results of the study. The same risks of bias are present in previously conducted in vitro and in vivo studies (before 2019) [93,94,95,96,97,98,99,100,101,102,103,104,105,106,107,108,109,110,111,112,113,114], meaning that more effort is needed to plan ideal, standardized protocols.

### 4.2. Chemical Agents as Preventive Products against Dental Caries

Following the principles of Minimal Invasive Dentistry, the current trends form a shift from restorative approaches to preventive approaches. The gold standard in dental caries prevention is the use of fluoride dentifrices, varnishes, mouthwashes [115], and restorative materials that release fluoride over time [116]. Various forms of fluorides (silver diamine fluoride, sodium fluoride, sodium monofluorophosphate, amine fluoride, and stannous fluoride) applied in dental science have proven to be adequate preventive agents against the occurrence of dental caries [117]. Three mechanisms are responsible for the anticariogenic effect of fluoride: these mechanisms include the inhibition of tooth demineralization, the enhancement of tooth remineralization, and the intervention of fluoride on bacterial metabolic activity through stability disturbance of bacterial cell membranes. Different forms of chlorhexidine are also proposed as preventive strategies for dental caries [118]. It is well documented that the antimicrobial properties of chlorhexidine are associated with bacterial cell membrane disruption. In low concentrations, chlorhexidine affects the metabolic activity of bacteria and is bacteriostatic, while in higher concentrations, it shows a bactericidal perspective by initiating irreversible precipitation of the cellular content [119,120]. It is an effective antimicrobial at a 0.12 to 0.2% concentration and is used as a gold standard control in various studies. These chemical agents present, inter alia, several side effects such as fluorosis due to early exposure–overexposure to fluoride components and the staining of teeth, taste alteration, increased mineral uptake into biofilm, and calculus formation, as well as oral mucosa irritation through the constant use of chlorhexidine supplements [27,28,29,30]. The development of resistant microbial strains is an additional drawback of the use of synthetic drugs—chemical agents restricting in that manner their long-term application [120]. To overcome these side effects, researchers tend to focus their interest on the investigation of natural, biological compounds as an alternative approach in dental caries prevention. Either solely used or combined, herbs are proven to be safe and effective in the management of various oral diseases [121,122].

### 4.3. The Potential Mechanism of Action of Herbal Extracts against Cariogenic Bacterial Strains

Over the years, a growing interest in plants that are rich in natural antimicrobial compounds has been observed. Phytochemical analysis has revealed that most of the antibacterial substances in plants are secondary metabolites that have special physiological functions [123]. The potential mechanism of action of herbal plants might be attributed to chemical components in the plant’s structure, their mechanical cleaning ability (e.g., the mechanical cleaning ability of miswak), or both of these traits working simultaneously [124]. 

A prime example of this direction is the dental caries preventive ability of *Galla chinesis*, a natural traditional Chinese medicine. Phytochemical tests have confirmed the presence of gallotannins as the main anticariogenic component [125]. Gallic acid and methyl gallate present inhibitory effects on the growth of cariogenic and periopathogenic bacteria [126]. Methyl gallate exhibits antimicrobial activities through its antibiofilm adhesion ability and its repressive effect against extracellular matrix enzymes, as well as through the inhibition of oxidative phosphorylation (direct action on microbial metabolism) [125]. Gallic acid promotes cell membrane disruption and the subsequent leakage of cytoplasmic content [127]. Furthermore, phytochemicals of *Galla Chinensis* have a direct effect on glycosyltransferase activity [128], and gallotannins can inhibit the demineralization of enamel [126,129]. 

Licorice has been studied extensively for its anticaries properties [49,74,94,95,109,130,131,132,133,134,135]. Phytochemical analysis of the roots of *Glycyrrhiza uralensis* verified the presence of pterocarpanes (glycyrrhizol A and glycyrrhizol B), along with four isoflavonoids [134]. Glycyrrhizol A and one isoflavonoid presented the most abundant anticariogenic properties. Furthermore, glycyrrhizic acid may inhibit the *S. mutans* count and its acid production in a laboratory environment [49,136].

Further indicative examples of plant extracts that present evidence-based anticariogenic properties through adequate literature references are listed below:

*Acacia nilotica* contains alkakoids, saponins, cardiac glycosides, tannins, flavonoids, and anthraquinones [102]. These phytochemical constituents may be responsible for the antimicrobial and antifungal action of the plant extract. Further, eucalyptus is found to contain alkaloids, phenolic compounds, steroids, cardiac glycosides, and terpenes that exhibit antibacterial activity against *S. mutans* [137]. In a clinical study by Amornchat et al. in 2006, the use of a mouthwash containing *A. myriophylla* significantly reduced *S. mutans* counts in the saliva of schoolchildren [138]. This effect was attributed to a triterpenoid, four steroids, and three flavonoids. Further investigations revealed that lupinifolin (flavonoid) possessed the main anticariogenic role compared to the rest of the phytochemical compounds [100]. Moreover, the essential oil of *Carum copticum* contains thymol, which proved to be primarily associated with damaging effects on both the cellular cytoplasmic membrane and Adenosine Triphosphate (ATP) production [139]. Thymol has a significant effect on the lipid fraction of the microorganism plasma membrane, causing changes in membrane permeability and the leakage of intracellular materials [62,140]. Also, the medicinal effects of Triphala (polyherbal Ayurvedic medicine consisting of fruits of the plant species *Emblica officinalis*, *Terminalia bellerica*, and *Terminalia chebula*) are strongly correlated with the presence of chemical compounds such as flavonoids [56,68,77]. Triphala’s use in oral health maintenance and dental caries prevention is documented [141,142]. More precisely, it is found that an active member of Triphala, namely, *E. officinalis*, consists of phytochemicals that bind to bacterial cell wall proteins, leading to a reduction in the hydrophobic adherence of *Streptococcus mutans* to the tooth surface [143]. Additionally, chlorogenic acids are naturally occurring polyphenolic compounds found in green coffee bean extracts and are responsible for the significant reduction in *S. mutans* strains [107].

Several studies have indicated that polyphenols possess remarkable anticaries properties by targeting various aspects of oral health, including inhibiting the growth and virulence of cariogenic bacteria, modulating biofilm formation, and reducing acid production [144,145,146,147,148,149]. Representative research on this topic conducted by Ferrazzano et al. in 2011 provided insights into the anticariogenic properties of plant polyphenols. The study highlighted the ability of polyphenols to combat dental caries through their multifaceted mechanisms of action. These mechanisms involve interference with the growth and activity of cariogenic bacteria, thus inhibiting their ability to form biofilms and produce acids that contribute to enamel demineralization [144]. Moreover, a study by Ferrazzano et al. in 2016 investigated the in vivo release of quercetin polyphenol incorporated in chewing gum and its antibacterial activity. The results demonstrated the potential of polyphenols for promoting oral health by effectively inhibiting the growth of cariogenic bacteria and reducing the risk of dental caries [150]. Furthermore, the study conducted by Ferrazzano et al. in 2017 evaluated the in vitro antibacterial activity of pomegranate juice and peel extracts against cariogenic bacteria. The findings revealed significant antibacterial effects, suggesting the therapeutic potential of pomegranate-derived polyphenols in preventing dental caries [151]. Studies by Kong et al. in 2022 and Guo et al. in 2023 highlighted the homeostasis maintenance, disease prevention, and therapeutic applications of polyphenols in oral health. These studies emphasized the diverse biological activities of polyphenols, including their anti-inflammatory, antioxidant, and antimicrobial properties, which collectively contribute to their beneficial effects on oral health [42,146]. 

The most dominant and anticariogenic chemical compound of green tea is catechin and, more precisely, epigallocatechin gallate (EGCG). Several studies have provided evidence supporting the efficacy of green tea extracts, particularly EGCG, in preventing dental caries [152,153,154,155]. This chemical compound exhibits a multifaceted mechanism of action, since it inhibits the growth and virulence of cariogenic bacteria. It effectively interferes with cell membrane integrity, and it suppresses the expression of genes involved in acid production and biofilm formation by *S. mutans* [42]. Furthermore, EGCG exhibits potent antioxidant properties, which contribute to its anticaries effects by reducing oxidative stress and inflammation in the oral cavity [151]. EGCG’s ability to scavenge free radicals and modulate inflammatory pathways helps maintain oral homeostasis and prevent caries formation [146]. In addition to its direct antimicrobial and antioxidant effects, EGCG has been shown to interfere with the quorum sensing mechanisms of cariogenic bacteria, disrupting their ability to communicate and coordinate virulence factor expression [42]. This mechanism further attenuates the pathogenic potential of these bacteria and reduces their ability to form biofilms, which are critical for the development of dental plaque and caries lesions [152]. 

All in all, the inhibition of Adenosine Triphosphate (ATP) production or energy metabolism, the increase in cell membrane permeability, alterations in pH homeostasis, cell morphology deterioration, cytoplasmic deregulation, enamel remineralization enhancement, and the direct inhibition of biofilm formation and biofilm adherence are the presumptive anticariogenic mechanisms of herbal biological extracts. The performed analytical methods, such as high-performance liquid chromatography coupled with mass spectrometry (HPLC-MS), demonstrate that the presence of several phytocompounds, such as alkaloids, flavonoids, steroids, tannins, and phenolic compounds, is responsible for the antimicrobial effect of herbal extracts. 

## 5. Considerations on Plant-Derived Compounds for Caries Prevention

It should not be forgotten that a plethora of mouthwashes containing herbal extracts are available on the market and have been briefly studied by researchers. It is found that commercially available herbal mouthwashes that contain naturally occurring products such as red ginseng extracts, chamomile, ocimum, and echinacea, as well as tea tree oil, peppermint, or aloe vera, present comparable antibacterial effects to commercially available mouthwashes containing chlorhexidine [156] Overall, although it is well documented that various plant extracts contain bioactive compounds with anticaries properties, such as polyphenols, further research is needed to validate their clinical efficacy [44]. Future research designs should include longitudinal clinical studies with standardized protocols to evaluate the long-term clinical efficacy and safety of natural compounds in diverse populations. These studies would provide valuable insights into the effectiveness of herbal extracts in preventing dental caries over extended periods, thereby establishing evidence-based guidelines for clinical use. Moreover, exploring innovative delivery systems for natural compounds could enhance their therapeutic potential and facilitate their integration into routine oral care practices [2]. Mouthwashes, chewing gum, dental varnishes, and other novel formulations offer convenience in delivering bioactive herbal compounds into the oral cavity. The standardization of extracts is also crucial for ensuring consistency in the composition and potency of natural compounds used in clinical trials and oral care products. However, standardization is complex due to the diverse chemical compositions of natural extracts and variations in extraction methods. Moreover, a variability in study methodologies poses challenges in comparing the efficacy of different natural compounds across clinical trials. Variations in participants’ demographic characteristics, study designs, outcome measures, and intervention protocols can influence the interpretation of research findings. To address this issue, researchers should focus on guidelines, such as the Consolidated Standards of Reporting Trials (CONSORT), to enhance the reproducibility and reliability of study results. Additionally, post-marketing surveillance and pharmacovigilance efforts are essential to monitor the real-world use of natural compounds in clinical settings and detect any emerging safety concerns.

## 6. Conclusions

Collaborative efforts among researchers, clinicians, policymakers, and industry stakeholders are essential to advance the field of natural compounds for caries prevention. With an interdisciplinary collaboration and knowledge exchange, stakeholders can address existing challenges, such as the standardization of compounds, the variability in study methodologies, and limited long-term data, thereby accelerating the transition from research designs to clinical implementation. Continued investment in research and innovation is crucial to unlock the full potential of natural compounds in promoting oral health and preventing dental caries. Overcoming these challenges, researchers can develop natural compounds as preventive agents for dental caries, ultimately improving oral health outcomes for individuals worldwide and providing sustainability to human ecosystems.

## Figures and Tables

**Table 1:** In vitro studies presented in literature based on herbal extracts and their preventive capability on dental caries.

Study/Year	Objective	Types of Specimens/Type of Control Group	Tests	Bacterial Strains	Conclusions
Malvania et al., 2019 [49]	Determination of the activity of licorice root extract on *Streptococcus mutans* in comparison to chlorhexidine and fluoride mouthwash.	Experimental groups: different concentrations of aqueous and ethanolic licorice root extracts Positive control groups (a) Chlorhexidine (CHX) mouthwash (b) Fluoride mouthwash	Agar diffusion method → zone of inhibition assessment Broth microdilution method → minimum inhibitory concentration (MIC) determination	*S. mutans*	Mean zones of inhibition of chlorhexidine mouthwash, fluoride mouthwash, aqueous and ethanolic licorice root extracts against *S. mutans* at 24 h are 23 mm, 14.2 mm, 15.8 mm, and 22.4 mm, respectively. The minimum inhibitory concentrations of aqueous and ethanolic licorice root extract on *S. mutans* are 20 mg/mL and 12.5 mg/mL, respectively. The antibacterial effect produced by ethanolic licorice root extract on *S. mutans* was comparable to chlorhexidine mouthwash, while significantly higher in comparison with the aqueous form and fluoride mouthwash.
Aloha et al., 2019 [50]	To determine the antibacterial and antifungal activities of *Eurycoma Longifolia Jack* (*Tongkat Ali*/E.L) root extract.	Experimental group: E.L. root ethanol extract of 200 mg/mL (soxhlet method) Positive control groups: nystatin and ampicillin Negative control group: 25% ethanol	Agar disk diffusion assay → zone of inhibition determination Broth microdilution method →MIC determination	*S. mutans*, *Lacticaseibacillus casei* (former name *Lactobacillus casei*), *Candida albicans*	E.L extract inhibited the growth of *C. albicans* and *S. mutans* at a concentration of 200 mg/mL with zones of inhibition of 16.0 ± 3.0 mm and 7.0 ± 1.0 mm, respectively. There was no antimicrobial effect of the extract on *Lactobacillus casei*. The zone of inhibition of *S. mutans* is 7.0 ± 1.0, and it is smaller than the positive control (ampicillin), which is 31.0 ± 0.50. MIC of ethanol extracts of E.L. against *S. mutans* is found to be at 25 mg/mL.
Zeng et al., 2019 [51]	Assess the effectiveness of quercetin and kaemferol of *Nidus Vespae* (honeycomb of *Polistes Olivaceous*, *P. Japonicus Saussure*, and *Parapolybiavaria Fabricius*) against *S. mutans* biofilm formation.	Experimental group: quercetin and kaemferol of Nidus Vespae Control group: chlorhexidine 0.12%	Compound identification → high-performance liquid chromatography–photodiode array–electrospray source ionization–multistage mass spectrometry (HPLC-PDA-ESI-MS) analysis Microdilution assay MIC determination minimal biofilm inhibition concentration 50% (MBIC50) minimal biofilm reduction concentration 50% (MBRC50) Inhibition of *S. mutans* biofilm formation examined using: in vitro biofilm model and confocal laser scanning microscopy (CLSM), scanning electron microscopy (SEM), Colony-Forming Unit/mL counting (CFU/mL), pH measurement, biofilm dry weight determination, total protein measurement, viable cells measurement, insoluble and soluble glucans formation	*S. mutans*	Quercetin and kaemferol demonstrated a comparable capability of *S. mutans* killing in biofilms, compared to chlorhexidine.
Mahalakshmi et al., 2019 [52]	Antimicrobial properties of *Solanum xanthocarpum* and *Pistacia lentiscus* extracts on cariogenic oral microbial flora.	Experimental groups Aqueous extracts of *S. xanthocarpum* Aqueous extracts of *P. lentiscus* In a standard concentration and varying dilutions, separately evaluated Positive control group: chlorhexidine	Agar diffusion method for zone of inhibition assessment Microdilution method for MIC determination	*S. mutans*, *Lactobacillus* species, and Actinomyces viscosus	All the tests with the standard concentration of the extracts (neat) produced a zone of inhibition, whereas further dilution of the herbal extracts did not produce any zone of inhibition. Both herbal products possess statistically significant antimicrobial properties. The antimicrobial effects of the herbal extracts were almost on par with commercially available allopathic agents like chlorhexidine. (Statistically significant differences.) No significant difference in antimicrobial efficacy between *S. xanthocarpum* and *P. lentiscus* on the test group of bacteria.
Oluwasina et al., 2019 [53]	Antimicrobial potential of a herbal toothpaste.	Experimental groups: Different toothpastes are formulated from ethanol extracts of *Syzygium aromaticum*, Dennettia tripetala and *Jatropha curcas latex*, either solely or in combination. Control groups: (a) 3 commercial fluoride toothpastes (b) Commercial antibiotics such as fucloxacillin and ketoconazole (c) Distilled water	Agar well diffusion method for zone of inhibition assessment Microdilution method for MIC determination Phytochemical analysis by gas chromatography/mass spectrometry pH determination of toothpaste	*Escherichia coli*, *Bacillus* sp., *Staphylococcus aureus*, *Staphylococcus epidermidis*, *Micrococcus luteus*, *S. mutans*, *Streptococcus pyogenes*, *Lactobacillus acidophilus*, *C. albicans*	The formulated toothpastes have a better and significant (*p* < 0.05) antimicrobial effect when compared to commercial toothpastes. Phenols, flavonoids, alkaloids, and saponins are present: *S. aromaticum*: eugenol (83.58%), caryophyllene (4.35%) and phenol, 2-methoxy-4-(2-propenyl)-, acetate (12.07%), *D. tripetala*: glutaric acid (57.57%), eugenol (2.9%), caryophyllene (1.12%), and 1,6,10-dodecatrien-3-ol, 3,7,11-trimethyl-,(E)- (3.36%)
Alshahrani et al., 2020 [54]	To identify the effects of nicotine exposure on the inhibitory effects of cinnamon water extract on *S. mutans* biofilm formation.	Experimental group: cinnamon aqueous extract in broth with varying nicotine concentrations Control group: broth without cinnamon aqueous extracts.	A preliminary experiment was conducted to determine the MIC and the minimum biofilm inhibitory concentration (MBIC) of cinnamon water extract alone on the growth of *S. mutans* in Tryptic soy broth supplemented with 1% sucrose (TSBS). *S. mutans* culture with varying nicotine concentrations (0–32 mg/mL) in Tryptic soy broth supplemented with 1% sucrose (TSBS) with or without a standardized concentration (2.5 mg/mL) of cinnamon aqueous extract Spectrophotometer to determine total growth absorbance and planktonic growth Crystal violet dye and absorbance measurement for biofilm formation determination	*S. mutans*	Cinnamon was able to inhibit biofilm formation significantly (*p* < 0.05). The presence of 2.5 mg/mL cinnamon water extract inhibits nicotine-induced *S. mutans* biofilm formation from 34 to 98% at different concentrations of nicotine (0–32 mg/mL).
Rai et al., 2020 [55]	Evaluate and compare the anticariogenic properties of different plant extracts against various cariogenic microorganisms.	Experimental groups: ethanol extracts of *Ocimum sanctum* (Tulsi), *Terminalia chebula* (Harad), *Tinospora cordifolia* (Guduchi), and *Glycyrrhiza glabra* (licorice) No control group	Agar diffusion test for zone of inhibition assessment Polarized light microscope for decay depth assessment	*S. mutans* and *L. acidophilus*	*Glycyrrhiza glabra* (licorice) extract had potent antibacterial efficacy against *S. mutans* and *L. acidophilus*. *T. chebula* produced a less inhibitory effect and more decay depth when compared to *G. glabra* (licorice) and *O. sanctum* (Tulsi) and more inhibitory zones and less decay depth of microbial growth than *T. cordifolia* against *S. mutans* and *L. acidophilus* studied at all time intervals.
Ramalingam et al., 2020 [56]	The antimicrobial efficacy of a mixed herbal powder extract (MHPE) against cariogenic microorganisms was investigated.	Experimental group: mixed herbal powder extract (MHPE) of *A. arabica* (bark), *Terminalia chebula* (fruits), *Terminalia bellerica* (fruits), and *Emblica officinalis* (fruits) (*Triphala*) Positive control group: chlorohexidine digluconate 0.12% Negative control group for biofilm studies: micro plates with media without inoculum	Agar diffusion method for zone of inhibition assessment Microdilution method for determination of MIC, Minimum Bactericidal Concentration (MBC) kinetics of killing, biofilm disruption, and anticaries effect of MHPE (live/dead staining biofilm assay, CLSM, SEM evaluation, continuous-flow biofilm model)	*S. mutans*, *L. casei*, *A. viscosus* and *C. albicans*	MHPE exhibited inhibition zones ranging from 12.5 to 24.0 mm. The highest inhibition zone was recorded at a concentration of 50 mg/mL. MIC for *S. mutans* was between 12.23 and 36.7 mg/mL MBC values ranged from 36.7 to 110.65 mg/mL. The inhibitory concentration of MHPE was three-fold higher than CHX. A significant reduction in cell count (49–95%) was observed with an increasing time and higher concentrations.
Zhang et al., 2021 [57]	Influence of Ligustrum robustum extract (LRE) on the biofilm formation of *S. mutans* and the mechanism of its action, as well as identifying its chemical components.	Experimental group: traditional Chinese herbal tea extract (*Ligustrum robustum* extract) Positive control group: xylitol	Phytochemical analysis HPLC-MS and nuclear magnetic resonance (NMR) Antimicrobial activity **confocal laser scanning microscopy (CLSM)** for quantification of bacteria and exopolysaccharide (EPS) synthesis **Crystal violet stain** for quantitative measurement of *S. mutans* biofilm formation **CFU counting** for assessment of inhibitory activity of LRE on *S. mutans* biofilm **zymogram assay** for the effect of the extract on the enzymatic activity of gtfs **qRT-PCR (Real-Time Polymerase Chain Reaction)** for relative expression levels of comD, come, and gtf genes *S. mutans* microstructure assessment treatment with LRE was investigated both on glass coverslips and ex vivo bovine dental enamel by **scanning electron microscopy (SEM).** Biocompatibility assessment of LRE **CCK-8 test** on human oral cells **MIC, MBC, MBIC** of LRE on different *S. mutans* strains.	*S. mutans* and 8 clinical isolates + *S. mutans* glucosyltransferase-encoding genes gtfB, gtfC, and gtfD, and the quorum sensing (QS) factors comD and comE	Phytochemicals identified: (1) Ligurobustoside B, (2) Ligurobustoside N, (3) Ligurobustoside J, and (4) Ligurobustoside C. *L. robustum* extract could reduce *S. mutans* sucrose-dependent adhesion and inhibited the subsequent biofilm formation LRE inhibits *S. mutans* EPS synthesis LRE inhibits the virulence of gene expression and the enzymatic activity of Gtf proteins in *S. mutans*. The reduced expression of comC, comD, and comE by LRE may cause decreased biofilm formation as well as reduced survivability for *S. mutans*. LRE had a greater antimicrobial effect than xylitol.
Herdiyati Y. et al., 2021 [58]	*Basil* (*Ocimum americanum*—Lamiaceae family) fatty acid as an antimicrobial agent against oral bacteria.	Experimental group: lauric acid of *ocimum americanum* Positive control groups: chlorhexidine and fosfomycin	Structural characterization of lauric acid of *ocinum americanum* nuclear magnetic resonance spectrometer FT-IR spectrometer mass spectrometry antimicrobial analysis microdilution method for determination of MIC/MBC	*S. mutans* and *Streptococcus sanguinis*	Lauric acid showed the potential as a new natural antibacterial agent through MurA inhibition in bacterial cell wall biosynthesis. Lauric acid was more active against *S. sanguinis* as compared to *S. mutans*. Chlorhexidine presented a similar effect to lauric acid.
Zayed et al., 2021 [59]	Isolate *S. mutans* from different oral samples taken from saliva and dental plaque specimens and determine their capability for biofilm formation. Evaluate the antibiofilm activity of aqueous and alcoholic green tea extracts.	A total of 150 isolates were recovered from seventy-five dental plaque samples (dps) and seventy-five saliva samples (ss) collected from patients having different degrees of dental caries. Experimental groups: Two green tea extracts (aqueous and alcoholic) were tested for their antibiofilm formation activity against some selected *S. mutans* isolates. Control group: wells containing non-inoculated media	Determination of antibiofilm activity of aqueous and alcoholic green tea extracts using microtiter plate assay Minimum biofilm inhibitory concentration (MBIC) is determined	*S. mutans* isolates (ten isolates recovered from dental plaque specimens and another ten recovered from saliva samples)	The prepared alcoholic green tea extract was observed to show antibiofilm activity at a lower concentration than that of the aqueous extract. The alcoholic extract of green tea exhibited antibiofilm activity against the strong biofilm-producer isolates at concentrations of 3.1, 6.3, 12.5 mg/mL, and the aqueous extract of green tea exhibited antibiofilm activity at concentrations 6.3, 12.5, 25 and 50 mg/mL.
Balhaddad et al., 2021 [60]	Investigate the antibacterial effect of *Salvadora persica* (*S.persica*) methanol and aqueous extracts against *S. mutans* biofilm.	Experimental groups: different concentrations of *S. persica* methanol or water extracts Negative control group: *S. mutans* overnight culture and TSB supplemented with 1% sucrose growth media Sterility control group: only TSB growth media	Tryptic soy broth (TSB) supplemented with 1% sucrose (microdilution broth assay) to determine MIC and bacterial growth (planktonic and biofilm growth) after optical density evaluation on a spectrophotometer	*S. mutans*	Minimum biofilm inhibitory concentration (MBIC) = 10 mg/mL. The *S. persica* aqueous extract did not demonstrate any biofilm inhibition at any concentration. The type of extract and its concentration are essential factors to achieve antimicrobial effectiveness with *S. persica*.
Adeleye et al., 2021 [61]	Incorporation of ethanol extract of *Massularia acuminata* (M.A.) twigs in a formulation of herbal toothpaste and evaluation of its antibacterial Activity, compared with a commercially available herbal toothpaste, against two dental pathogens.	Experimental groups: toothpastes containing 1%, 2%, 3%, 4%, and 5% M. acuminata ethanol extract Control groups: commercially available herbal toothpaste and gentamicin (positive control)	Sensory and physicochemical properties evaluation (phytochemical evaluation, p.H, moisture, foaming, viscosity determination) Agar well diffusion method for antibacterial properties evaluation (inhibition zone)	*S. aureus* and *S. mutans*	The phytochemical constituents present in the ethanol extract of the *M. acuminata* twig included anthraquinones, saponins, flavonoids, alkaloids, tannins, and flavonoids. pH range of 7.18–7.83 The antibacterial activity of the formulated toothpastes increased significantly with an increase in the extract concentration. The incorporation of *M. acuminata* extract in the formulation of herbal toothpaste prevented the growth of *S. aureus* and *S. mutans*.
Mehdipour et al., 2022 [62]	Antimicrobial and antibiofilm effects of three herbal extracts on *S. mutans* compared with chlorhexidine 0.2%.	Experimental groups: *Carum copticum*, *Phlomis bruguieri*, and *Marrubium parviflorum* methanolic extracts with various concentrations Control groups: chlorhexidine 0.2% and dimethyl sulfoxide	Antimicrobial activity well diffusion method and MIC determination Antibiofilm activity of the extracts microtitre plate method The inhibitory effect on biofilm formation was measured by the ELISA reader apparatus Crystal violet test for cell adhesion and biofilm formation	*S. mutans*	Although all of the methanolic herbal extracts can inhibit *S. mutans* growth and remove the biofilm, the effect of *Carum copticum* was better than *Phlomis bruguieri* and *Marrubium parviflorum* The antibiofilm activity of the three extracts was lower than the common 0.2% chlorhexidine mouthwash.
Lavaee et al., 2022 [63]	Evaluation of the antimicrobial properties of *Pimpinella anisum* and *Oregano* *Vulgare*.	Experimental groups: ethanolic and methanolic extracts of *Pimpinella anisum* and *Oregano vulgare* (separately as well as combined) Control group: chlorhexidine	MIC and MBC determination of ethanolic and methanolic extracts of *Pimpinella anisum* and *Oregano vulgare* by macrodilution and microdilution methods	*S. sanguinis*, *S. mutans*, *S. salivarius*	Hydroalcoholic extracts of *Pimpinella anisum* and *Oregano Vulgare* were effective antibacterial agents against pathogens. The combination of these two extracts showed the highest antibacterial properties on all the bacteria evaluated.
Jalil et al., 2022 [64]	Investigation of the antibacterial, antibiofilm, and antioxidative effect of *Piper betle* leaf extract.	**In vitro–in silico** Experimental group: chloroform extracts of *Piper betle* leaves Positive control group: piperacillin/tazobactum Negative control group: DMSO	2700 samples (2500 caries patients and 200 control patients and identification of bacterial isolates) Phytochemical analysis of chloroform extracts of Piper betle leaf: thin-layer chromatography analysis (TLC), FTIR analysis, GC-MS analysis Microdilution assay for MIC determination Inhibition zone assessment for antibacterial activity evaluation Time kill assay, SEM analysis, protein estimation by SDS-PAGE In silico development of PPI network and GO and KEGG pathway enrichment Analysis (gene ontology (GO) analysis and Kyoto Encyclopedia of Genes and Genomes (KEGG) enrichment analysis) Molecular docking analysis	*Bacillus gaemokensis*	Presence of several phytocompounds, such as alkaloids, flavonoids, steroids, tannins, and phenolic compounds. The antibacterial role of *P. betle* chloroform extract against *B. gaemokensis* is evident. Spirost-8-en-11-one,3-hydroxy(3β,5α,14β,20β,22β,25R), an oxosteroid in nature, was observed to exhibit remarkable antibacterial potential (12 mm) against *B. gaemokensis*. Bacterial cells treated with *P. betle* extract demonstrated decreased growth, while the extract was also observed to exhibit the inhibition of biofilm formation (70.11%) and demolition of established *B. gaemokensis* biofilms (57.98%). In silico network pharmacology analysis elucidated proteins like ESR1 and IL6 to be majorly involved in biological pathways of dental caries, which also interact with protective ability of *P. betle*. Molecular docking demonstrated the highest binding affinity of Spirost-8-en-11-one,3-hydroxy-,(3β,5α,14β,20β,22β,25R) with bacterial proteins FabI (−12 kcal/mol), MurB (−17.1 kcal/mol), and FtsZ (−14.9 kcal/mol).
AL-Qaralusi et al., 2023 [65]	Antibacterial activity of tea (black and green tea aqueous extracts) against salivary mutans-type Streptococci and an analysis of the effect of non-nutritive sweeteners on the antibacterial activity of these extracts against salivary *mutans*-type Streptococci.	Experimental groups: Different concentrations of black and green tea aqueous extracts (50 mg/mL, 100 mg/mL, 200 mg/mL, 300 mg/mL, and 500 mg/mL), two types of non-nutritive sweeteners used: stevia in different percentages and sucralose in different percentages Negative control group: agar with microbial inoculums without the addition of the extract Positive control group: agar with different concentrations of tea extracts without microbial inoculums	Agar well diffusion technique for assessing the antimicrobial activity of both types of tea extracts and the antibacterial potential of stevia and sucralose	*S. mutans* isolates	At a dosage of 225 mg/mL for black tea extracts and 200 mg/mL for green tea extracts, all mutans isolates were destroyed. It is found that increasing the content of nonnutritive sweeteners interfered with the antibacterial activity of black and green tea aqueous extract against salivary mutans-type Streptococci.
Govindaram et al., 2023 [66]	Antimicrobial properties of herbal extracts and their effects on human oral keratinocytes,	Experimental groups: *Carica papaya*, *Trachyspermum ammi*, *Caesalpinia crista linn* extracts Positive control groups: chlorhexidine	Microdilution method to determine the MIC MTT assay for cytotoxicity evaluation	*S. mutans*, *Lactobacillus* sp., and *A. viscosus*	The three test herbal extracts possess effective anticariogenic properties near to that of chlorhexidine, and *T. ammi* proved to be the most potent. The extracts at different concentrations also proved to be safe and non-cytotoxic.
Maria et al., 2023 [67]	Determine and analyze the minimum zone of inhibition of *Curcuma amada* against *S. mutans*.	Experimental groups: 5%, 10% and 25% concentration of C. Amada ethanolic extract Control group: 5%, 10%, 25% chlorhexidine 0.2%	Well diffusion method using blood agar plates and determination of minimum zone of inhibition	*S. mutans*	The antibacterial activity of *C. amada* against *S. mutans* raises the possibility of incorporating it into various dental therapeutic agents.
Kripalani et al., 2023 [68]	Comparative evaluation of the phytochemical analysis and efficacy of four plant-derived extracts against *S. mutans*.	Experimental groups: Emblica officinalis (Amla), Vitis vinifera seeds, Psidium guajava (*P. guajava*) Linn leaves, and Acacia nilotica (*A. nilotica*, Babul) extracts were prepared individually Control groups: not mentioned	Phytochemical analysis (Mayer test, Dragendorff test, Wagner test, Molish test, Salkowski test, Lieberman–Burchard test, Keller Kiliani test, Ninhydrin test, Millon’s test, Ferric chloride test, Spot test, Foam test, Saponification test) and bacterial growth analysis by CFU/mL counting	*S. mutans*	Alkaloids, carbohydrates, tannins, and flavonoids are present in all the extracts. Steroids and proteins are present in *P. guajava* Linn. Proanthocyanidins were present in Vitis vinifera. Saponins and anthraquinones were present in *A. nilotica* exclusively. There is no bacterial fermentation in *Emblica officinalis* and *Vitis vinifera*, and a minimum amount of growth in *P. guajava Linn* and *A. nilotica*.
Nehavarshini et al., 2023 [69]	Formulation of a nanoemulsion, combined with aqueous extracts of herbal powders, and testing its efficiency as a caries-preventing mouthwash.	Experimental group 1: nanoemulsion of gingelly oil, neem oil, clove oil, and peppermint oil, Polysorbate 20 Experimental group 2: nanoemulsion of gingelly oil, neem oil, clove oil, and peppermint oil, Polysorbate 20 + *Acacia arabica*, *Terminalia chebula*, *Terminalia bellerica*, and *Emblica officinalis* Positive control group: chlorhexidine Negative control group: ultra-pure water	Broth microdilution method for MIC determination Biofilm adherence test by 2% sucrose addition and crystal violet staining Biofilm assay with microtiter plates Artificial Mouth assay by the use of human tooth samples, DIAGNOdent pen, and non-static CDC bioreactor	*S. mutans*, *L. casei*, *A. viscosus*	The nanoemulsion with plant extract showed anti-adherence and anti-bioflm activity and hence can be used as a potent anticariogenic mouthwash. Bioflms of *S. mutans*, *L. casei*, *A. viscosus*, and combinations were inhibited by nanoemulsion with herbal extracts more than simple nanoemulsion and chlorhexidine. The antimicrobial effects of plant extract *(E. ofcinalis*, *T. chebula*, *T. bellerica*, and *A. arabica*) and nanoemulsion (gingelly oil, neem oil, clove oil, and peppermint oil) are observed in combination against caries-causing bacteria.
Karnjana et al., 2023 [70]	Determination of the effects of extracts of *Streblus asper*, *Cymbopogon citratus*, *Syzygium aromaticum* and the formulation of green synthesized silver nanoparticles (AgNPs) on *S. mutans* growth and biofilm formation.	Experimental groups: aqueous and ethanolic extracts of *S. asper*, *C. citratus*, *S. aromaticum*, and a mix of the three herbs Positive control group: 0.2% chlorhexidine Negative control group: disk of 10% DMSO	Phytochemical profile by high-performance liquid chromatography coupled to high-resolution mass spectrometry (HPLC–MS) Fetermination of antibacterial activities against *S. mutans* and antibiofilm formation (broth microdilution method, agar disk diffusion assay) → inhibition zone + MIC + MBC determination Microbial adhesion to hydrocarbon (MATH test) for cell-surface hydrophobicity of S. mutans measurements. Molecular docking technique Morphological observation of *S. mutans* biofilms by scanning electron microscopy (SEM)	*S. mutans*	The ethanolic extracts of *S. asper*, *S. aromaticum*, and *C. citratus* could be used as natural alternative agents, with multiple actions against *S. mutans* infections, as they exhibited antibacterial activities. The formulated AgNPs from the ethanolic extracts could enhance the antibacterial activities of the plant extracts. Evident interaction between luteolin isolated from *C. citratus* and glucosyltransferase protein (GtfB) → promising antibiofilm activity. Significant decrease in the biofilm area of the AgNPs treated. Vanillin, 3,3′-methylene-bis(4-hydroxybenzaldehyde), and palmitic acid were found in *S. asper*. Gallic acid, biflorin, quercetin, kaempferol, eugenol, rhamnocitrin, 2,3,4-trimethoxyacetophenone, and copaene were identified from *S. aromaticum*. Five metabolites were found in C. citrates, including kaempferol, β-caryophyllene oxide, luteolin, β-vatirenene, and isocaryophyllene. The compounds that were found in each of the ethanolic extracts were also found in the toothpaste formulated. These included gallic acid, chlorogenic acid, quercetin, luteolin, rhamnetin, quercetin 3′ -O-glucuronide, kaempferol, β-vatirenene, 5-βH-16β-hydroxylkamaloside, and palmitic acid.
Falakdin et al., 2023 [71]	Investigation of the antimicrobial activity of aerial parts of *Scrophularia striata* (*S. striata*) and the galls of *Quercus infectoria* (*Q. infectoria*) against cariogenic microorganisms.	Experimental groups: A. Hydroalcoholic extracts of *S. striata* and *Q. infectoria* (evaluated separately and combined) B. Fabrication of a herbal mouthwash after determination of the MIC, MBC and Fractional Inhibitory Concentration Index (FICI) by adding propylene glycol and the investigation of stability and tannic acid content for 60 days Control groups: inoculated and un-inoculated broths	Microdilution method according to the Clinical and Laboratory Standards Institute (CLSI) guidelines for MIC determination and MBC determination solely for *Q. infectoria* and *S. striata* against 3 cariogenic microorganisms. Checkerboard method for assessing the combined antimicrobial effect of the two herbal extracts by fractional inhibitory concentration index (FICI) calculation The herbal mouthwash was investigated for pH stability, flavor, taste, color, antimicrobial activity, phytochemical characteristics, and stability (time kill assay → LIVE/DEAD cells, HPLC for tannic acid determination) t0: immediately after the fabrication of the mouthwash t1: after 60 days of storage	*S. mutans*, *Streptococcus obrinus*, *C. albicans*	*Q. infectoria* gall extract possesses efficient antimicrobial activity that was synergistically enhanced in the presence of *S. striata* extract. Mouthwash prepared using these extracts showed desirable organoleptic characteristics, antimicrobial activity, and stability. Extracts of S. striata and *Q. infectoria* galls can be used together for preparing dental products with effective anticariogenic properties. Positive synergistic effects of *S. striata* and *Q. infectoria* gall extracts on growth inhibition and eradication of cariogenic microorganisms.
Bollamma et al., 2023 [72]	Assessing the potential antimicrobial activity of various *Robusta coffee* extracts on *S. mutans*.	Experimental groups: Extracts of Green coffee bean Coffee pulp Coffee leaves prepared by various methods Boiling Maceration Soxhlet extraction using different solvents: i. Distilled water ii. Ethanol iii. Ethyl acetate iv. Hexane to determine the extraction procedure which gives adequate antimicrobial action against *S. mutans*. Control group: not mentioned	MIC determination by microdilution methods Inhibitory zones assessment	*S. mutans*	Antimicrobial properties against *S. mutans* could not be established for green coffee bean extract and coffee leaf. Coffee pulp extracts using ethyl acetate and ethanol showed clear zones of inhibition in well cultures on *Mitis salivarius* agar enriched with bacitracin. The yield was greater when ethanol was used as a solvent. MIC was 12.5 mg/mL. Coffee pulp is a potential herbal alternative for caries prevention, considering its antimicrobial action against *Streptococcus mutans*.
Takada et al., 2024 [73]	Investigation of the inhibitory effects of 15 medicinal herbs on causative bacteria for dental caries and periodontal disease.	Experimental groups: aqueous extracts of 15 medicinal herbs Control group: non-treated control medium	Bacterial growth and biofilm formation were assessed using the broth microdilution method The extract of the herb *Hibiscus sabdariffa* (hibiscus) was analyzed using HPLC MIC determination of hibiscus extract	*S. mutans*, *S. sobrinus*, *Aggregatibacter actinomycetemcomitans*, *Porphyromonas gingivalis*, *Prevotella intermedia*	Hibiscus exerted a significant inhibitory effect on all the oral pathogenic bacterial strains. The pigment delphinidin-3- sambubioside, which is found in hibiscus extract, was identified as a particularly important inhibitory component.

**Table 2:** Presentation of in vivo, in situ, and ex vivo clinical studies that evaluate the preventive potential of herbal extracts against dental caries.

Study/Year	Objective	Types of Specimens/Type of Control Group	Tests	Bacterial Strains	Conclusions
Chen et al., 2019 [74]	Investigate the efficacy of a herbal lollipop containing licorice for reducing salivary *S. mutans* levels, and investigate its impact on salivary microbiome.	**In vivo study with in vitro background** 37 high-risk children with salivary *S. mutans* levels > 5 × 10^5^ cells/mL, determined by antibody-based method, were enrolled in study Experimental group: two lollipops a day (one in the morning after brushing teeth, and one at night, at least 30 min before brushing teeth) for three weeks Control group: no lollipops	In vitro project Broth microdilution method for MIC determination Optical density measurements and CFU/mL counting for bacterial growth and kill assessment Real-time PCR for quantification of *S. mutans* and total bacteria In vivo project Unstimulated saliva collection (t0 = baseline, t1 = 1 week, t2 = 2 weeks, t3 = 3 weeks) and oral microbiome assessment by 16S rRNA gene sequencing	*S. mutans* and the whole oral microbiome	Licorice extract displays targeted killing against *S. mutans* without affecting the biodiversity of the community. For high caries-risk children aged 3–6, daily use of 2 licorice-containing lollipops for 3 weeks significantly reduced salivary *S. mutans* levels compared to the control group. Salivary microbiome analysis showed either no change or an increase in the phylogenetic diversity of the oral community following herbal lollipop usage.
Khoramian Tusi et al., 2020 [75]	The effect of antimicrobial activity of a mouthwash containing *Teucrium polium* on Oral *S. Mutans*.	**A randomized cross-over clinical trial study** 22 volunteers divided into 2 groups Group A: using mouthwash with *T. polium* for 2 weeks Group B: control group using mouthwash without *T. polium* for 2 weeks 3 weeks washout period Group A became Group B Group B became Group A *S. mutans* of saliva was measured t baseline: before t 2: after each phase to compare effects of mouthwashes	The TYCSB (tryptone–yeast–cysteine–sucrose–bacitracin) medium of *S. mutans* was formed and CFU/mL counting took place	*S. mutans*	There was no statistical difference between the numbers of *S. mutans* colonies per one milliliter of saliva in the groups before using the mouthwashes. When the mouthwash containing *Teucrium polium* was used, there was a significant decrease in the number of *S. mutans* colonies.
Kalpavriksha et al., 2021 [76]	Evaluate and compare the antibacterial effect of glass ionomer cement (GIC) containing CHX and miswak extract on Streptococcus mutans and *Streptococcus sobrinus* in Early Childhood Caries (ECC) children.	**Clinical study** 45 children with ECC divided into 3 treatment groups GIC with CHX GIC with miswak Control group (GIC + deionized water) Plaque sample collection t1: before cavity preparation t2: 1 month after treatments t3: 3 months after restoration	Polymerase chain reaction analysis (qRT-PCR)	*S. mutans* and *S. sobrinus*	No statistically significant difference between group I (CHX) and group II (miswak) in *S. mutans* and S. *sobrinus* count at 1-month and 3-month intervals. Statistically significant difference in *S. mutans* and *S. sobrinus* count between group I (CHX) and group III (control) and group II (miswak) and group III (control) at 1-month and 3-month intervals. 1% chlorhexidine digluconate and aqueous extract of miswak are equally effective against *S. mutans* and *S. sobrinus*. Miswak can be used as an alternative herbal antimicrobial that can be incorporated in anhydrous GIC.
Bane et al., 2022 [77]	Evaluate the antibacterial efficacy of *Emblica officinalis* lollipops on *S. mutans* counts and pH levels in institutionalized visually impaired children.	**A double-blinded, randomized, interventional clinical study** 60 institutionalized visually impaired children Experimental group: *E. officinalis* lollipop Control group: placebo lollipop Volunteers subjected to the respective lollipops twice daily for seven days t baseline: before lollipop t1: 7 days after lollipop consumption	Collection of stimulated saliva at baseline and after 7 days Microbial assay and pH level determination Microdilution method and CFU/mL counting Single electrode digital pHmeter	*S. mutans*	A nearly 30.65% reduction in Streptococcus mutans count was obtained at the end of seven days in the *E. officinalis* group, while it was only 5.90% in the placebo group. The efficacy of the study group (*E. officinalis* lollipop) in inhibiting the *S. mutans* count was better than the control group (placebo lollipop) A significant increase in the pH level in the experimental group is to be seen.
Gunther et al., 2022 [78]	Antimicrobial effects of *Rosmarinus officinalis* extract against oral microorganisms within in situ initial oral biofilms.	**In situ–ex vivo study** in situ biofilm samples (2 h) on bovine enamel from six healthy volunteers wearing oral splints were treated ex vivo with *R. officinalis* extract at concentrations of 20 mg/mL and 30 mg/mL. Experimental group: *R. officinalis*-treated biofilms at (a) 20 mg/mL (b) 30 mg/mL concentrations Positive control: 0.2% CHX on the bovine enamel slabs Negative untreated control: NaCl 2nd negative untreated control: dimethyl sulfoxide	High-performance thin-layer chromatography for the analysis of *R. officinalis* extracts Colony-forming units counting for viable bacterial cells calculation MALDI-TOF (matrix-assisted laser desorption/ionization coupled to time-of-flight mass spectrometry) and biochemical testing for surviving bacterial identification Live/dead staining and epifluorescence microscopy for visualization and quantification of initial biofilm	Total oral microbiome	The number of colony-forming units in the *R. officinalis*-treated biofilms was significantly lower than in the untreated controls. The CFUs were comparable to the CFUs yielded by CHX-treated biofilms. Large intra- and interindividual bacterial variability was observed. Except for *Campylobacter* sp, the average amount of all bacterial taxa was lower after treatment with *R. officinalis* than in the untreated biofilms. A total of 49 different species were detected in the untreated bioflms, while only 11 bacterial species were detected in the *R. officinalis*-treated biofilms. Live/dead staining confirmed that the *R. officinalis*-treated biofilms had significantly lower numbers of surviving bacteria than the untreated biofilms. The treatment with *R. officinalis* extract has a significant potential to eliminate microbial oral initial biofilms.
Bollamma et al., 2023 [79]	This study aims to assess the potential antimicrobial activity of *Robusta coffee* pulp extracts on *S. mutans*.	**In vivo** 39 participants were divided into three groups with 13 participants each Group A (negative control), sterile water; Group B (positive control), 0.2% chlorhexidine mouth rinse; Group C, 2.5% coffee pulp extract mouthwash (prepared according to minimum inhibitory concentration). Saliva samples collection at T0 = baseline T1 = 1 h- post rinse T2 = 2 weeks after rinsing	The mouthwash was prepared at a concentration of 25 mg/mL, which is a 2.5% concentration with 5% dimethyl sulfoxide (to improve the solubility of the extract in distilled water) *S. mutans* colony count at three time intervals using image-based software analysis	*S. mutans*	The coffee pulp mouth rinse and positive control showed a statistically significant reduction in the microbial count at 2 weeks post-rinse period, compared to the negative control group. The difference in the microbial count reduction at 2 weeks post-rinse period was not statistically significant between the coffee pulp mouth rinse and positive control. The mean microbial count did not differ significantly across the three different time intervals in the negative as well as positive control groups, but differed significantly in the coffee pulp extract-based mouth rinse. A major drawback seen in the case of coffee pulp mouth rinse was its bitter taste, which could not be masked. It could reduce patient compliance. The coffee pulp extract-based mouth rinse is a potential anticariogenic agent.

## Data Availability

Not applicable.
